# Role of Dectin-1 in peripheral nerve injury

**DOI:** 10.3389/fncel.2022.810647

**Published:** 2022-07-28

**Authors:** Angela Yu-Huey Hsu, Sung-Tsang Hsieh

**Affiliations:** ^1^School of Medicine, National Taiwan University, Taipei, Taiwan; ^2^Department of Medical Education, National Taiwan University Hospital, Taipei, Taiwan; ^3^Department of Anatomy and Cell Biology, National Taiwan University, Taipei, Taiwan; ^4^Graduate Institute of Brain and Mind Sciences, National Taiwan University, Taipei, Taiwan; ^5^Center of Precision Medicine, National Taiwan University Hospital, Taipei, Taiwan; ^6^Department of Neurology, National Taiwan University Hospital, Taipei, Taiwan

**Keywords:** Wallerian degeneration, macrophage, peripheral nerve injury, peripheral nerve regeneration, Dectin-1

## Abstract

Dectin-1, a C-type lectin receptor, plays a role in nerve injury in the central nervous system. However, whether it plays a role in the peripheral nervous system is not well understood. Our study showed the expression of Dectin-1 on the membrane of macrophages. We also used a sciatic nerve crushing injury model to demonstrate that there was a delay in nerve degeneration-related processes such as breakdown of injured myelinated nerve fibers and formation of myelin ovoid in groups injected with whole glucan particle soluble (WGPS), a Dectin-1 antagonist. There were also fewer intraneural blood vessels in the Dectin-1 antagonist treated group. Our study suggested inhibiting Dectin-1 delayed debris clearance, nerve degeneration, and angiogenesis after peripheral nerve injury.

## Introduction

Injuries to the peripheral nerve result in consequent processes denoted as Wallerian degeneration. One of the first steps of Wallerian degeneration is the clearance of injured axons by macrophages, followed by the regeneration of axons (Gaudet et al., 2011). Morphologically, myelinated nerves first undergo swelling, and the debris of degenerated myelinated nerves is later cleared to make way for the growth of regenerated myelinated nerves more densely packed and smaller in diameter than intact myelinated nerves (Guilbaud et al., 1993). Macrophages, recruited to the injury site as early as 3 days and peaking at 7 days after peripheral nerve injury, play an essential role in the clearance of nerve debris (Chen et al., 2015).

Two major groups of macrophages could participate in the events after peripheral nerve injury, namely the pro-inflammatory M1 group and the pro-regeneration M2 group (Kigerl et al., 2009). Dectin-1, a C-type lectin receptor that acts as a pattern recognition receptor of β(1,3)-glucans mainly on fungal cell walls, is expressed on the M2 macrophages (Martinez et al., 2006; Yamasaki, 2016). Moreover, Dectin-1 expression was also found in microglia of the central nervous system (Krasemann et al., 2017; Ye et al., 2020) and macrophages of the peripheral nervous system (Wang et al., 2020).

Previous studies have shown that stimulating Dectin-1 increased nerve injury and axon degeneration after spinal cord injury (Gensel et al., 2015) but promoted axon regeneration in an optic nerve crush model (Baldwin et al., 2015). Another study suggested that injecting apoptotic neurons into mice’s brains induced “homeostatic” microglia to express ApoE, TREM-2, and Dectin-1, promoting inflammation in the brain, linking the pathology of neurodegenerative diseases (Krasemann et al., 2017). The blockade of Dectin-1 and its downstream effector Syk decreased the brain infarct volume and improved functional neurological outcomes 3 days after ischemic stroke (Ye et al., 2020). Moreover, in a model of experimental autoimmune encephalomyelitis, Dectin-1 limits autoimmune neuroinflammation (Deerhake et al., 2021). These findings suggest that Dectin-1 played a role in neural regeneration and homeostasis in the central nervous system.

Moreover, some C-type lectin receptors, which have similar structures and functions as Dectin-1, played a role in the generation of neuropathic pain (Dominguez et al., 2014; Ishikawa et al., 2019). However, whether Dectin-1 mediates peripheral nerve degeneration, regeneration, or neuropathic pain remains unclear. This study applied a model of sciatic nerve crushing injury to study: (1) the expression of Dectin-1 in the peripheral nervous system; (2) the effect of Dectin-1 on peripheral nerve degeneration; and (3) the effect of Dectin-1 on functional outcomes after peripheral neuropathy.

## Materials and Methods

**Figure 1:**
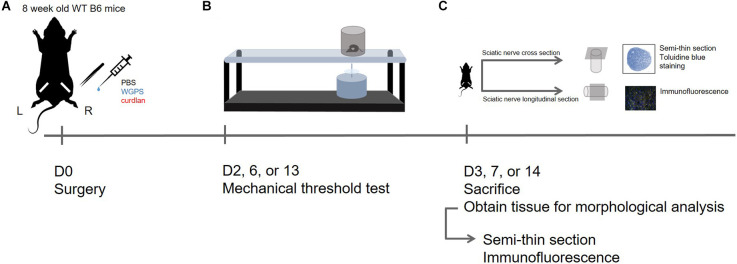
Experimental design in this study. **(A)** D0: Sham surgery was performed on the left sciatic nerve; sciatic nerve crush surgery and injection of PBS (vehicle control), WGPS (Dectin-1 antagonist), or curdlan (Dectin-1 agonist) was performed on the right sciatic nerve. **(B)** D2, 6, or 13: Mechanical threshold test was performed to assess functional recovery. **(C)** D3, 7, or 14: The animals were sacrificed to obtain sciatic nerves for morphology analysis. Semi-thin sciatic nerve cross-sections were stained with toluidine blue to assess myelinated nerve and blood vessel profile; immunofluorescence staining was done on longitudinal sections of the sciatic nerve to assess Dectin-1 expression and macrophage density.

The experimental design of this study is shown in Figure 1.

### Animals

Surgeries were performed on adult male C57BL/6J mice (8–9 weeks old). After sciatic nerve crushing surgery, animals were sacrificed at 3, 7, and 14 days after surgery to obtain sciatic nerve tissue for morphology analysis, and mechanical threshold tests were performed 1 day before the sacrifice (2, 6, and 13 days after surgery) of each group. All procedures were approved by the Institutional Animal Care and Use Committee, National Taiwan University College of Medicine, and were conducted according to the Guide for the Care and Use of Laboratory Animals from the National Research Council.

### Animal surgery

#### Sciatic nerve crush surgery

Animals were gas anesthetized by isoflurane, both thighs were shaved, and the skin of the right thigh was incised. The fascial plane between the gluteus maximus and the anterior head of the biceps femoris was opened to reveal the sciatic nerve. No. 5 forceps (Ideal-tek, Balerna, Switzerland) were dipped with carbon, and then the sciatic nerve was crushed for 30 s. The skin incision was closed with the EZ clip wound closing kit (Stoelting Co., Wood Dale, IL).

#### Sham surgery

The contralateral (left) sciatic nerve was exposed and mobilized but left intact and not crushed.

#### Sciatic nerve injection

The sciatic nerve was exposed as previously described. Immediately after nerve crushing injury, a 32 gauge needle (Hamilton robotics, Reno, NV) was carefully inserted into the nerve to 1 mm distal to the crush site. Four microliter of either phosphate buffer saline (PBS), solution of whole glucan particle soluble (WGPS 1 mg/ml in filtered ddH_2_O; Invivogen, San Diego, CA), or curdlan (25 mg/ml; Sigma-Aldrich, Saint Louis, MO), prepared as previously described (Baldwin et al., 2015) was injected into the nerve.

### Mechanical threshold test

Mechanical threshold tests were performed 1 day before sacrifice for each group. Mice were individually placed in a Plexiglas^TM^ container on metal mesh and allowed to habituate to the new environment. A mechanical stimulus was delivered to the plantar surface of the hind paw from below the floor of the test chamber with a dynamic plantar aesthesiometer (Ugo Basile, Comerio-Varese, Italy). A steel rod (diameter: 0.5 mm) was pushed against the hind paw with ascending forces of 0–50.0 g over 20 s. When the animal withdrew its hind paw, the mechanical stimulus stopped automatically, and the force at which the animal withdrew its paw was recorded to the nearest 0.1 g, which indicated the nociceptive threshold; each hind paw was alternatively tested five times with a minimal interval of 5 min between measurements. Due to the high variance within each group, the mechanical threshold ratio, i.e., the average mechanical threshold of the ipsilateral side divided by the average mechanical threshold of the contralateral side, was used for analysis.

### Tissue preparation

Animals were anesthetized and perfused intracardially first with 1% sodium nitrite solution (1 ml/g), then with 4% paraformaldehyde solution (2 ml/g). Tissues were post-fixed for another 2 h, then stored in 0.1 M phosphate buffer at 4°C. Before sectioning, tissues were cryoprotected in 30% sucrose in 0.1 M phosphate buffer. Tissues were cut into sections 8 μm in thickness with a cryostat (Leica, Wetzlar, Germany), then mounted onto gelatin-coated slides.

**Table 1:** List of primary antibodies.

**Target protein**	**Number**	**Company**	**Titration**
Dectin-1	AF1756	R and D Systems, Minneapolis, MN, United states	1:500
Iba1	019–19741	Wako, Osaka, Japan	1:250
CD206	GTX42263	Genetex, Taiwan, R. O. C	1:200

### Immunofluorescence staining (IF)

**Figure 2:**
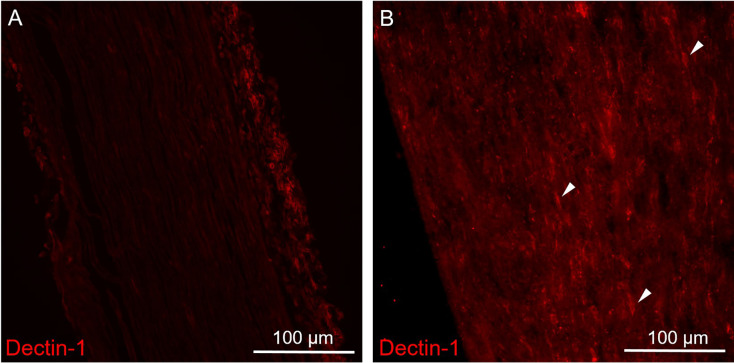
Immunofluorescence staining of Dectin-1 on the control (contralateral) and crushing injury (ipsilateral) side of the sciatic nerves. Longitudinal sections from mice sciatic nerve 7 days after surgery of the control (contralateral) side **(A)** and the side of crushing injury (ipsilateral) **(B)** were stained with anti-Dectin-1 primary antibody (Cy3). **(A)** There was no Dectin-1 expression on the control side. **(B)** After crushing injury, there was presence of cigarette-shaped positive staining areas mimicking the morphology of macrophage on the ipsilateral sciatic nerve (arrows).

Slides were washed with PBS for 5 min three times, then incubated in 0.5% Triton in PBS for 30 min. Sections were then incubated in primary antibodies listed below in Table 1 for 20–22 h. After rinsing in PBS, sections were incubated in secondary antibodies for 1.5 h (Alexa Fluor 488 conjugated donkey-anti-rabbit IgG, Cy3 conjugated donkey-anti-goat IgG, Alexa Fluor 488 conjugated donkey-anti-rat IgG, all from Jackson ImmunoResearch, West Grove, PA). Sections were then incubated in DAPI (Sigma-Aldrich, St. Louis, MO) solutions for 3 min, then rinsed with water and mounted in glycerol gelatin. The images in Figure 2 were acquired with a Zeiss Axio Imager M1 microscope. All other immunofluorescence images were acquired with a Zeiss Cell Observer spinning disk confocal microscope.

### Quantification of macrophage on myelinated nerves

Crushing injury sites with carbon labeling were recognized, and 400× tile photos were taken from 500 μm proximal to 500 μm distal to the injury site with a Zeiss Cell Observer spinning disk confocal microscope. Three slides randomly chosen, with sections at least 20 μm apart, were used for each mouse. The average of the three sections of each mouse was used for statistical analysis.

Cell numbers were calculated using Metamorph. Only cells with nuclei and positive staining were included in the analysis. Cell density was derived and expressed as the number of cells per square micrometer of the sciatic nerve (cells/μm^2^). Macrophage was defined by Iba1(+) cells; Dectin-1(+) macrophage was defined by [Dectin-1(+)/Iba1(+)] cells; Dectin-1(+) M2 macrophage was defined by [Dectin-1(+)/CD206(+)] cells.

### Semi-thin section of sciatic nerves

The preparation of the semi-thin sections of sciatic nerves followed our established protocol (Chiang et al., 2005). The sciatic nerves were collected from 1 mm proximal to the crushed site to the site of trifurcation. The most distal 2 mm were taken and then fixed in 5% glutaraldehyde in 0.1 M phosphate buffer at 4°C for 2 h. The tissues were post-fixed in 2% osmic acid for 2 h at room temperature, dehydrated, and embedded in Epon 812 resin (Polysciences, Philadelphia, PA). One micrometer thick semi-thin sections were cut on a Reichert Ultracut E ultramicrotome (Leica, Wetzlar, Germany) and stained with toluidine blue.

### Quantifying myelinated nerve fibers and blood vessels

Myelinated nerve fibers were photographed under a Leica DM2500 microscope. All myelinated nerve fibers in the entire fascicle were counted first with the AxonSeg software (Zaimi et al., 2016). False negatives were then manually corrected using the Image-Pro PLUS software (Media Cybernetics, Silver Spring, MD). Myelinated nerve fiber, myelin ovoid, and intraneural blood vessel density were derived and expressed as the number of nerve fibers, myelin ovoids, or intraneural blood vessels per square micrometer of nerve fascicle (number of objects/μm^2^).

Structures with the following features were defined as “myelinated nerve fibers”: homogenous, light-colored axons surrounded by darker, continuous myelin sheath of the same thickness with clear outlines. “Myelin ovoids” were structures of the following characteristics: round and homogenous objects as dark as myelin. “Intraneural blood vessels” were structures with the following features: lumen surrounded by at least one endothelium cell with apparent nuclei.

### Statistical analysis

Kruskal-Wallis test with post-hoc Mann-Whitney test was used to analyze the median of myelinated nerves due to its skewed distribution. ANOVA with *post-hoc* *t*-test was used to analyze myelinated nerve density, myelin ovoid density, blood vessel density, macrophage density, macrophage ratio, and difference in mechanical thresholds. GraphPad Prism 7 was used for statistical analysis.

## Results

### Increased expression of Dectin-1 on the sciatic nerve 7 days after crushing injury

To explore the expression patterns of Dectin-1, we performed immunofluorescence staining on the sciatic nerves. There was no Dectin-1 expression in the contralateral sciatic nerve (Figure 2A). In the ipsilateral sciatic nerve distal to the crushed site, there was an increased expression of Dectin-1(+) cells with the morphology mimicking macrophages (Figure 2B).

### Dectin-1 was localized on the macrophage cell membrane in the sciatic nerve

**Figure 3:**
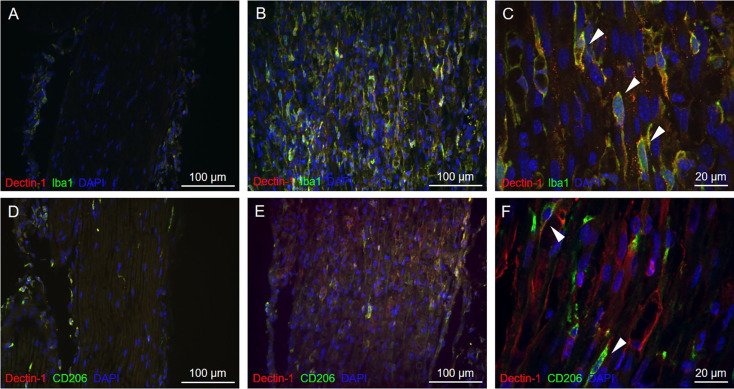
Immunofluorescencedouble staining of Dectin-1 and macrophage markers of thecontralateral and crushed side. Longitudinal sections from thecontralateral **(A)** and crushed **(B,C)** sciatic nerve7 days after crushing injury were immunostained withanti-Dectin-1 antibody (Cy3) and anti-Iba1 antibody (AlexaFluor 488); longitudinal sections from the contralateral **(D)**and crushed **(E,F)** sciatic nerve 7 days after crushing injurywere immunostained with anti-Dectin-1 antibody (Cy3) andanti-CD206 antibody (Alexa Fluor 488). **(C)** Higherresolution images in 630× showed the presence of Dectin-1(+)macrophages (arrows). **(F)** Higher resolution images in630× showed the presence of Dectin-1(+) M2 macrophages (arrows).

To understand whether Dectin-1 was expressed on macrophages, we performed double-labeling immunofluorescence staining of Dectin-1 and Iba1, a pan-macrophage marker. On the ipsilateral sciatic nerve distal to the crush site, Dectin-1 was colocalized with Iba1, indicating the expression of Dectin-1 on the macrophage cell membrane (Figures 3B,C). However, on the contralateral side, there was nearly no Dectin-1 expression or Dectin-1(+) Iba1(+) cells (i.e., Dectin-1 positive macrophages; Figure 3A).

To determine if Dectin-1 is expressed on the M2 subset macrophages, we performed double-labeling immunofluorescence staining of Dectin-1 and CD206, an M2 macrophage marker. On the ipsilateral side, Dectin-1 was colocalized with CD206 (Figures 3E,F), indicating the presence of Dectin-1(+) M2 macrophages distal to the crush site after injury. On the contralateral side, there were nearly no Dectin-1 positive M2 macrophages (Figure 3D).

### Temporal change in nerve morphometry after crushing injury

**Figure 4:**
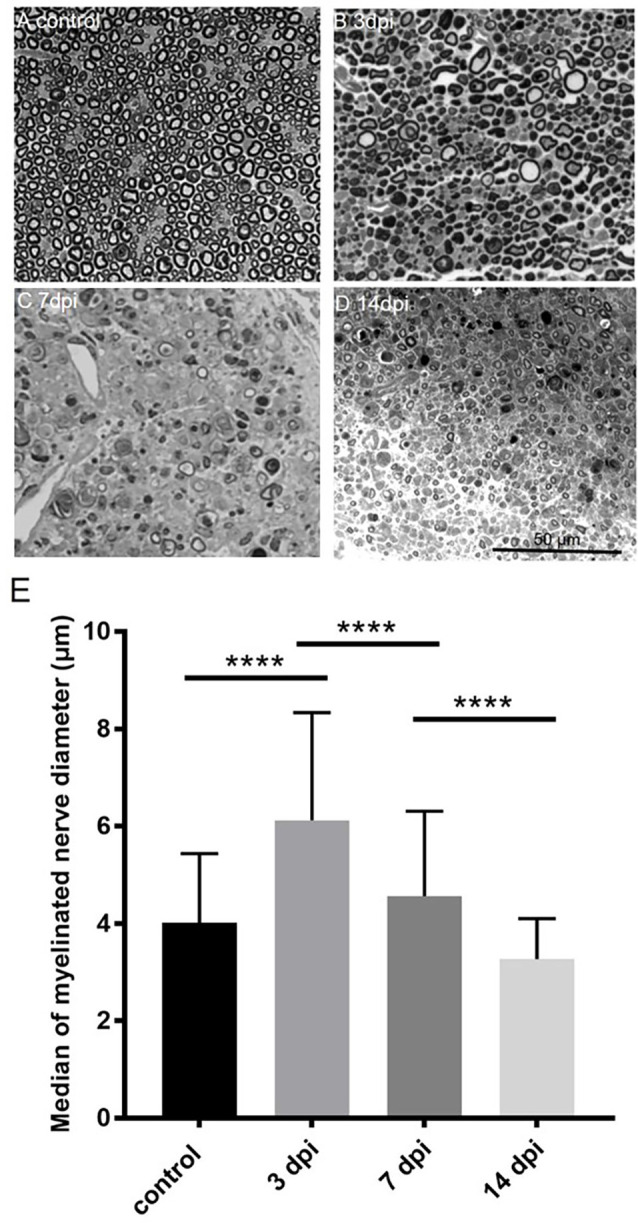
Temporalchange of semi-thin sections in the PBS injected group(ipsilateral side) and intact sciatic nerve (contralateral side). Semi-thin cross-sections 1 μm in thickness of the crushedsciatic nerve injected with PBS distal to the injury site were stained with toluidine blue. The representative figures are from **(A)** intact sciatic nerve of sham surgery (contralateral). **(B)** Ipsilateral sciatic nerve 3 days after crushing injury. **(C)** Ipsilateral sciatic nerve 7 days after crushing injury. **(D)** Ipsilateral sciatic nerve 14 days after crushing injury. **(E)** Medians of myelinated nerve diameter from different groups. Data are represented as median ± SD (*n*: control = 7, 3 dpi = 4, 7 dpi = 3, 14 dpi = 3), ****p < 0.0001, dpi: day post-injury. Data obtained from the sham surgery (contralateral) side was denoted as “control” in this figure. Compared with intact myelinated nerves of the control group, the median of myelinated nerve diameter increased in 3 days after crushing injury but decreased in 7 and 14 days after crushing injury.

To determine myelinated nerve morphology after crushing injury, we analyzed sciatic nerve semi-thin sections at 3, 7, and 14 days after nerve injury. We then examined the temporal patterns of the nerve morphometry according to the myelinated nerve profiles. The median of myelinated nerve diameter was increased 3 days after crushing injury, then continued to decrease up to the end of the experiment, i.e., 14 days after crushing injury (Figure 4).

### Effects of Dectin-1 on post-crushing nerve morphometry

To investigate the effects of Dectin-1 on nerve degeneration and regeneration, we conducted interventional experiments by intraneural injection of the following reagents after sciatic nerve crushing: (1) PBS as the vehicle control; (2) WGPS as the Dectin-1 antagonist; and (3) curdlan as the Dectin-1 agonist.

**Figure 5:**
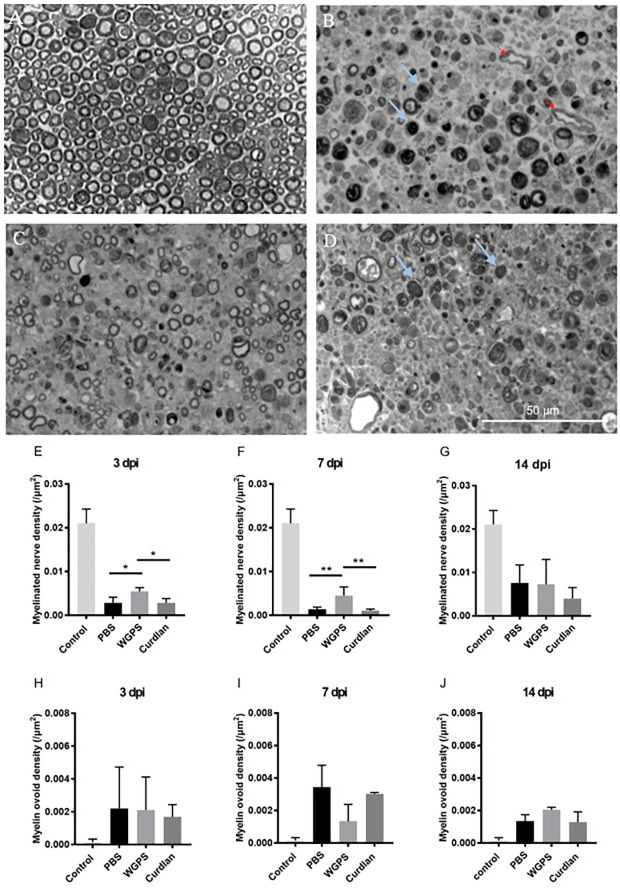
Semi-thinsection morphometry analysis of myelinated nerve and myelin ovoid.Semi-thin cross-sections 1 μm in thickness of the crushedand injected sciatic nerve distal to injury were stained withtoluidine blue for morphometry analysis. The representative figures are from: **(A)** intact sciatic nerve of sham surgery (contralateral), **(B)** crushing injury plus PBS injection7 days after injury (ipsilateral), **(C)** crushing injury plus WGPS injection 7 days after injury (ipsilateral) and **(D)** crushing injury plus curdlan injection 7 days after injury (ipsilateral). Quantitative analysis of myelinated nerve density isshown in **(E–G)**. Quantitative analysis of myelin ovoid density is shown in **(H–J)**.** (A)** Most myelinated nerves were intact on the sham surgery (contralateral) side. **(B–D)** There were fewer intact myelinated nerves, more blood vessels (red arrows), and a trend of more myelin ovoid (blue arrows; *p* = 0.06) 7 days after injury. ** (E)** In the PBS and curdlan injected groups, there were fewer intact myelinated nerves 3 days after injury. Data are expressed as mean ± SD (*n*: control *n* = 7, 3 dpi: PBS *n* = 4, WGPS *n* = 3, curdlan *n* = 4; 7 dpi: PBS *n* = 6, WGPS *n* = 3, curdlan *n* = 3; 14 dpi: PBS *n* = 3, WGPS *n* = 3, curdlan *n* = 3), *p < 0.05. dpi, day post-injury. Data obtained from the sham surgery (contralateral) side was denoted as “control” in this figure. **p < 0.01.

We then quantified myelinated nerve density on semi-thin sections of the sciatic nerves to determine the effect of Dectin-1 on myelinated nerve morphometry. Representative image of the intact sciatic nerve is shown in Figure 5A. The myelinated nerve density of the WGPS group was significantly higher than that of the curdlan and PBS groups 3 and 7 days after crushing injury (Figures 5B–F). There was no significant difference in myelinated nerve density among the three groups 14 days after crushing injury (Figure 5G).

Since myelin ovoid is an indicator of complete myelinated nerve degeneration (Tricaud and Park, 2017), we quantified the density of myelin ovoids. There was no difference in myelin ovoid density among the three groups 3 days after crushing injury (Figure 5H). However, there was a trend of lower myelin ovoid density in the WGPS group than in the PBS group 7 days after crushing injury (*p* = 0.06, Figure 5I). There was no difference in myelin ovoid density among the three groups 14 days after crushing injury (Figure 5J).

**Figure 6:**
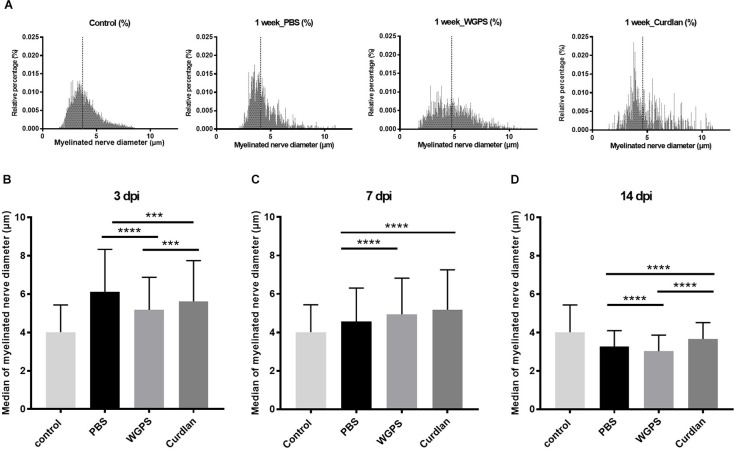
Histogramand median of myelinated nerve density.** (A)** Histogram of myelinated nerve diameter distribution percentage from semi-thin section morphometry analysis 7 days after crushing injury in the sham (contralateral), PBS (ipsilateral), curdlan (ipsilateral), and WGPS (ipsilateral) groups. Dotted lines denote the median of each group. **(B–D)** Medians of myelinated nerve diameter from different groups. **(B)** The median of myelinated nerve diameter was the smallest in the WGPS injected group 3 days after the crushing injury. **(C)** The median of myelinated nerve diameter was greater in the WGPS and curdlan groups than in the PBS group 7 days after crushing injury. **(D)** The median of myelinated nerve diameter is the smallest in the WGPS injected group 14 days after crushing injury. Data are represented as median ± SD (*n*: control *n* = 7, 3 dpi: PBS = 4, WGPS = 3, curdlan = 4; 7 dpi: PBS = 6, WGPS = 3, curdlan = 3; 14 dpi: PBS = 3, WGPS = 3, curdlan = 3), ***p < 0.001, ****p < 0.0001, dpi: day post-injury. Data obtained from the sham surgery (contralateral) side was denoted as “control” in this figure.

At 3 days after injury, the median of myelinated nerve diameter was smallest in the WGPS group compared with the PBS and the curdlan groups. At 14 days after injury, the median of myelinated nerve diameter was smallest in the WGPS group compared with the PBS and the curdlan groups (Figure 6).

### Effects of Dectin-1 on the intraneural vasculature of injured nerve

**Figure 7:**
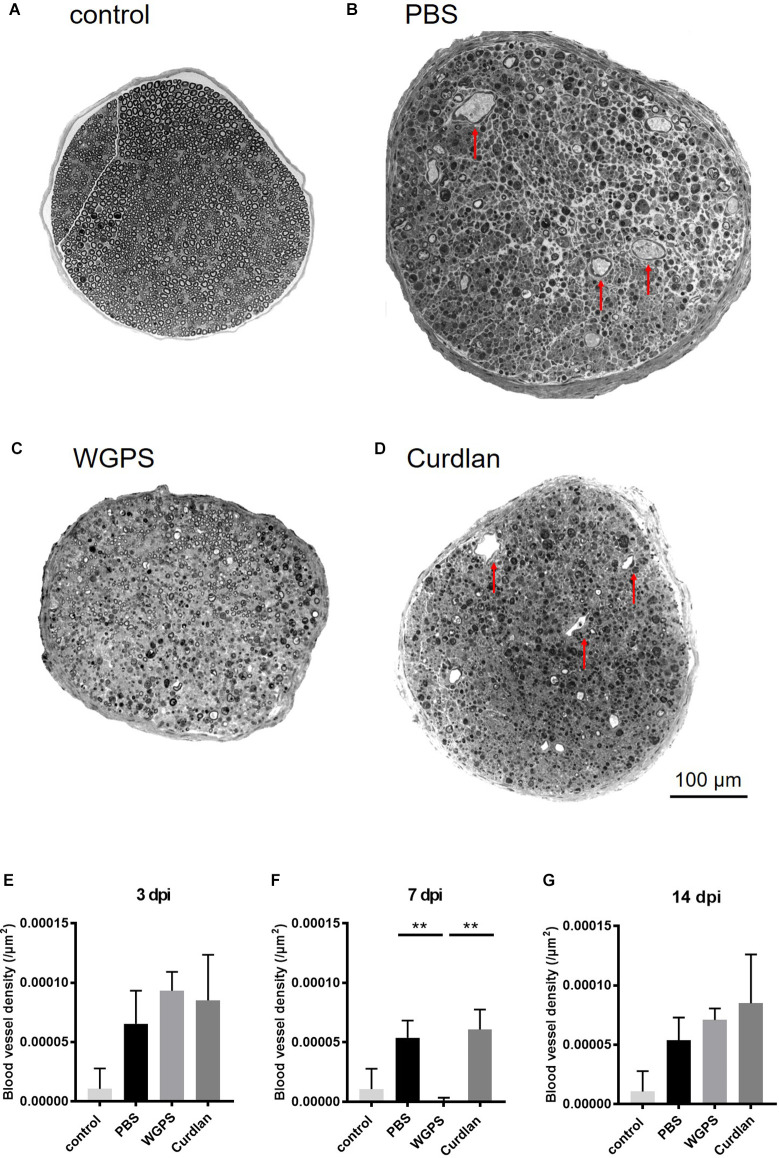
Semi-thinsection morphometry analysis of intraneural blood vessels. Semi-thin cross-sections 1 μm in thickness of the crushed and injected sciatic nerve distal to the injury were stained with toluidine blue for morphometry analysis. The representative figures are from **(A)** intact sciatic nerve of sham surgery (contralateral). **(B)** PBS injected group 7 days after crushing injury (ipsilateral). **(C)** WGPS injected group 7 days after crushing injury (ipsilateral). **(D)** Curdlan injected group 7 days after crushing injury (ipsilateral). Arrows indicate blood vessels. Quantitative analysis of intraneural blood vessel density is shown in **(E–G)**. There was a significant decrease in blood vessel density in the WGPS group 7 days after crushing injury than in the PBS and curdlan groups. Data are represented as median ± SD (*n*: control *n* = 7, 3 dpi: PBS = 4, WGPS = 3, curdlan = 4; 7 dpi: PBS = 5, WGPS = 3, curdlan = 3; 14 dpi: PBS = 3, WGPS = 3, curdlan = 3), **p < 0.01, dpi: day post-injury. Data obtained from the sham surgery (contralateral) side was denoted as “control” in this figure.

To explore the effect of Dectin-1 on angiogenesis, which is related to nerve regeneration, we quantified the intraneural blood vessel density of the sciatic nerves on semi-thin sections. The intraneural blood vessel density was lower in the WGPS group than in the PBS and curdlan groups 7 days after crushing injury (Figure 7).

### Effects of Dectin-1 on macrophage phenotype

To determine Dectin-1(+) cell recruitment at different time points after injury, we quantified the number of Dectin-1(+) cell densities at 3, 7, and 14 days after crushing injury on the crushed nerve in groups injected with PBS, WGPS, and curdlan. We did not quantify the Dectin-1(+) cell number in the contralateral nerves because Dectin-1(+) cells were absent (Figures 3A,D).

**Figure 8:**
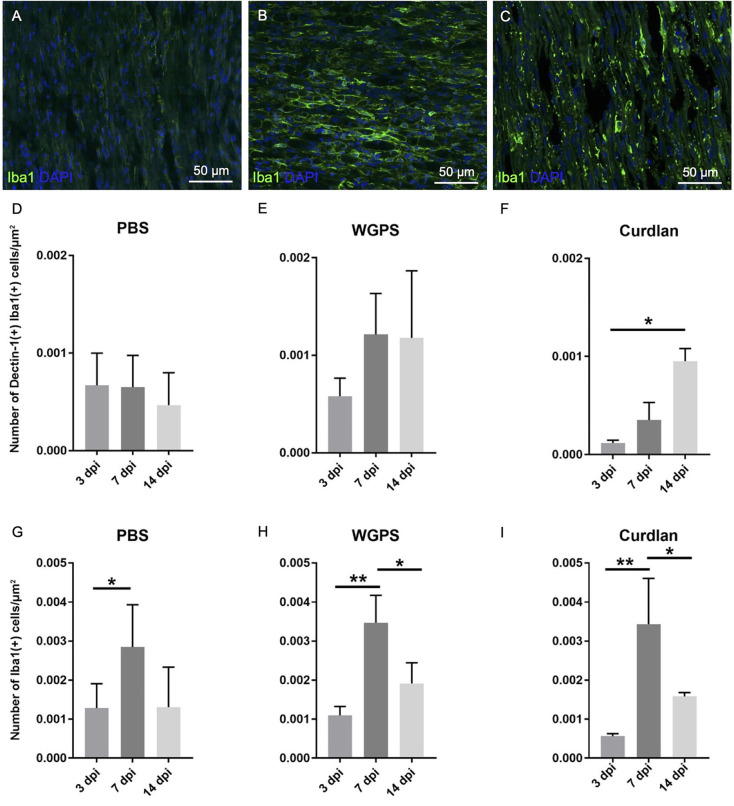
Analysis of Dectin-1 and Iba1 colocalization or Iba1 expression on crushed sciatic nerves. Longitudinal sections of the ipsilateral sciatic nerve from 500 μm proximal to 500 μm distal to the crushed site were stained with anti-Dectin-1 and anti-Iba1 antibodies or anti-Iba1 antibody only. Dectin-1(+) Iba1(+) cells or Iba1(+) cells were identified for analysis. **(A–C)** Representative figures from **(A)** crushing injury plus PBS injection 3 days after injury, **(B)** crushing injury plus PBS injection 7 days after injury, **(C)** crushing injury plus PBS injection 14 days after injury. **(D–F)** Quantitative analysis of Dectin-1(+) macrophage density [Dectin-1(+)/Iba1(+) cell density] at different time points. **(G–I)** Quantitative analysis of macrophage density [Iba1(+) cell density] at different time points. Macrophage density was significantly higher at 7 days after injury in the PBS, WGPS, and curdlan groups; there was no difference between Dectin-1 macrophage densities in the PBS and WGPS groups at different time points. There was increased Dectin-1 macrophage density in the curdlan group at 14 days compared with 3 days after injury. Data are represented as mean ± SD (*n*: 3 dpi: PBS = 4, WGPS = 3, curdlan = 4; 7 dpi: PBS = 5, WGPS = 3, curdlan = 3; 14 dpi: PBS = 3, WGPS = 3, curdlan = 3). **p < 0.01, *p < 0.05.

In the PBS group, the density of macrophages [Iba1(+) cell density] was greater at 7 days compared with 3 days after injury (Figure 8). In the WGPS and curdlan groups, the densities of macrophages were greatest at 7 days after injury (Figures 8H,I; and Supplementary Figure 2).

There were no differences between the densities of Dectin-1(+) macrophages [Dectin-1(+)/Iba1(+) cell density] at 3, 7, and 14 days after injury in the PBS and WGPS groups (Figures 8D,E; and Supplementary Figures 1A–F). In the curdlan groups, the density of Dectin-1(+) macrophages was significantly greater at 14 days compared with 3 days after injury (Figure 8F; Supplementary Figures 1G–I).

**Figure 9:**
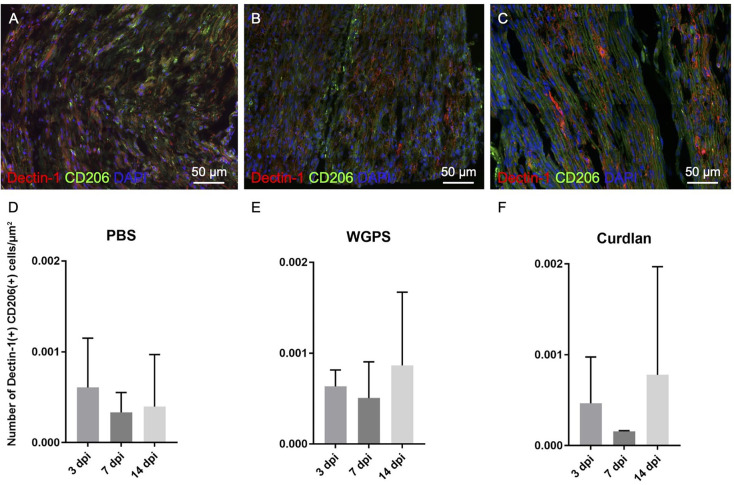
Analysis of CD206 and Dectin-1 colocalization on crushed sciatic nerves. Longitudinal sections of the ipsilateral sciatic nerve from 500 μm proximal to 500 μm distal to the crushed site were stained with anti-Dectin-1 and anti-CD206 antibodies. Dectin-1(+) CD206(+) cells were identified for analysis. **(A–C)** Representative figures from **(A)** crushing injury plus PBS injection 3 days after injury, **(B)** crushing injury plus PBS injection 7 days after injury, **(C)** crushing injury plus PBS injection 14 days after injury. **(D–F)** Quantitative analysis of Dectin-1(+) M2 macrophage density [Dectin-1(+)/CD206(+) cell density] at different time points. There were no difference between the groups. Data are represented as mean ± SD (*n*: 3 dpi: PBS = 4, WGPS = 3, curdlan = 4; 7 dpi: PBS = 5, WGPS = 3, curdlan = 3; 14 dpi: PBS = 3, WGPS = 3, curdlan = 3; 14 dpi: PBS = 3, WGPS = 3, curdlan = 3).

In all three groups at different time points, there were no differences between the densities of Dectin-1(+) M2 macrophages [Dectin-1(+)/CD206(+) cell density] (Figure 9; Supplementary Figure 3).

### Effects of Dectin-1 on mechanical thresholds

**Figure 10:**
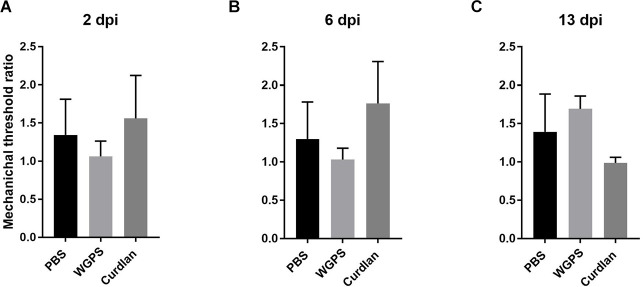
Mechanical nociceptive threshold ratio of the ipsilateral side and contralateral side. **(A)** 2, **(B)** 6, and **(C)** 13 days after crushing injury. Mechanical threshold tests were performed 1 day before sacrifice for each group. Mechanical threshold ratios were calculated as the average mechanical threshold of the ipsilateral side divided by the average mechanical threshold of the contralateral side. There was no differences between the groups. Data are expressed as mean ± SD (*n*: dpi 2: PBS *n* = 4, WGPS *n* = 3, curdlan *n* = 4; dpi 6: PBS *n* = 6, WGPS *n* = 3, curdlan *n* = 3; dpi 13: PBS *n* = 3, WGPS *n* = 3, curdlan *n* = 3). dpi, day post-injury.

To examine the effect of Dectin-1 on the functional outcomes after nerve crushing injury, we measured mechanical thresholds on both the surgical and contralateral sides 1 day before sacrificing the animals. The mechanical threshold of the ipsilateral side was increased compared to that of the contralateral side. Due to the high variance within each group, the mechanical threshold ratio, i.e., the average mechanical threshold of the ipsilateral side divided by the average mechanical threshold of the contralateral side, was used for analysis. However, there was no difference in the mechanical threshold ratio between any of the groups (Figure 10).

## Discussion

This study investigated the expression patterns and functional roles of Dectin-1 in the peripheral nervous system after nerve injury. We documented increased Dectin-1(+) macrophages after nerve crushing injury, which was also shown in previous reports (Wang et al., 2020). The increased Dectin-1(+) macrophages on the degenerating nerves also suggested the presence of endogenous ligands released from the nervous system after injury. Although we have not identified an endogenous ligand in our study, previous studies have identified endogenous ligands of Dectin-1 in various disease models and cell types, such as Galectin-9 in a model of experimental autoimmune encephalomyelitis (Deerhake et al., 2021), annexins on apoptotic cells (Bode et al., 2019), and vimentin in atherosclerosis (Thiagarajan et al., 2013). Future investigations are required to identify the nature of the endogenous ligand in our model.

Our study was the first to examine both the early and late processes of peripheral nerve degeneration under the same nerve injury model. Various studies performed on the central nervous system had implied that stimulating Dectin-1 promoted nerve degeneration at the early stages; other studies suggested that Dectin-1 activation promoted nerve regeneration at the later stages after nerve injury. However, these efforts applied different injury models on the central nervous system, focused on one certain time point after nerve injury, and had not examined the overall effect of Dectin-1 at different stages after nerve injury. For instance, inhibiting Dectin-1 activity reduced infarct volume and improved functional outcomes 3 days after ischemic stroke (Ye et al., 2020), while stimulating Dectin-1 activity decreased axon density 3 days after spinal cord injury (Gensel et al., 2015) but promoted optic nerve regeneration 14 days after optic nerve crush injury (Baldwin et al., 2015).

This report showed that Dectin-1 aided nerve degeneration and debris removal at the early stage of nerve injury. There was an increased myelinated nerve density and a trend of decreased myelin ovoid density in WGPS groups, suggesting inhibited degeneration of myelinated nerves and retained myelinated nerve profiles after inhibiting Dectin-1 activity.

Moreover, the initial response to nerve injury is calcium influx, axon swelling, and cytoskeleton breakdown (Gaudet et al., 2011; Villegas et al., 2014; Tricaud and Park, 2017). Therefore, the smallest median of myelinated nerve diameter in the WGPS group 3 days after crushing injury could indicate less inflammation and degeneration. After myelin debris was cleared, new fibers with a smaller diameter would appear and enlarge in size as they mature (Guilbaud et al., 1993; Ikeda and Oka, 2012). In our study, there was no difference between the myelinated nerve density in the WGPS, PBS, and curdlan groups 14 days after injury. Although not all myelinated nerves identified 14 days after nerve injury were regenerated fibers, the smallest myelinated nerve diameter of the WGPS group 14 days after crushing injury could still imply delayed maturation and enlargement of regenerated fibers. This is consistent with previous reports that myelin debris contains substances that might be harmful to nerve regeneration. Delayed clearance was associated with a delay in nerve regeneration-related processes such as maturation and enlargement of myelinated nerve diameters in the peripheral nervous system (Rotshenker, 2011).

Our results also implied that the inhibition of Dectin-1 could inhibit intraneural angiogenesis. Indeed, macrophages could secrete vascular endothelial growth factor (VEGF-A), promoting angiogenesis (Cattin et al., 2015). Therefore, based on our research, Dectin-1, located on macrophages, may participate in the events after nerve injury through phagocytosis and cytokine release by macrophages.

We did not observe a change in morphology, blood vessel density, or myelinated nerve fiber density between the curdlan and PBS groups. Since curdlan is associated with activation of macrophages (Rosas et al., 2008) and induce downstream immune responses and cytokine release, these difference might be on the molecular level and not present histopathologically at the observed time points after nerve injury. In the injured nervous system, endogenous ligands might be present for Dectin-1 (Krasemann et al., 2017; Deerhake et al., 2021). It is possible that the Dectin-1 pathway is already activated in our PBS injected group, and thus trying to activate it does not create significantly different effects morphologically.

In our crush injury model, the density of macrophages peaked at 7 days after injury, consistent with previous reports (Chen et al., 2015). The numbers of Dectin-1(+) macrophages are also similar across 3, 7, and 14 days except for the increase in Dectin-1 positive macrophages in the curdlan group at 14 days. Although previous studies indicated that curdlan is associated with polarization of M1 macrophage to the M2 subtype (Liu et al., 2019). There was no difference in macrophage polarization after intervention with Dectin-1 agonist and antagonist in our model.

We did not observe any difference in mechanical threshold ratios between different groups. A longer period of follow-up after the injury might be needed to observe any difference in functional recovery.

Our study suggested the presence of some endogenous ligands for Dectin-1 after peripheral nerve injury. Dectin-1 participated in debris clearance, myelin ovoid formation, and nerve degeneration in the earlier stage after nerve injury. Inhibiting Dectin-1 activity also hindered angiogenesis, implying the harmful effect of Dectin-1 inhibition in the events after peripheral nerve injury.

## Data Availability Statement

The raw data supporting the conclusions of this article will be made available by the authors, without undue reservation.

## Ethics Statement

The animal study was reviewed and approved by the Institutional Animal Care and Use Committee, National Taiwan University College of Medicine.

## Author Contributions

Methodology, resources, writing—review and editing, and project administration: AY-HH and S-TH. Formal analysis, investigation, data curation, and writing—original draft: AY-HH. Supervision and funding acquisition: S-TH. All authors contributed to the article and approved the submitted version.

## Funding

This work was supported by the Ministry of Science and Technology, Taiwan (107-3017-F-002-002 and 107-2320-B-002 -043 -MY3) and Ministry of Education (107L9014-2).
