# Supplementary material for: Role of Dectin-1 in peripheral nerve injury
Source: Front Cell Neurosci. 2022 Jul 28;16:810647. doi: 10.3389/fncel.2022.810647 (PMC9366223; doi:10.3389/fncel.2022.810647)
Supplement: Supplementary file 1 [file Data_Sheet_1.PDF]

Supplementary Material

1. Supplementary Figures

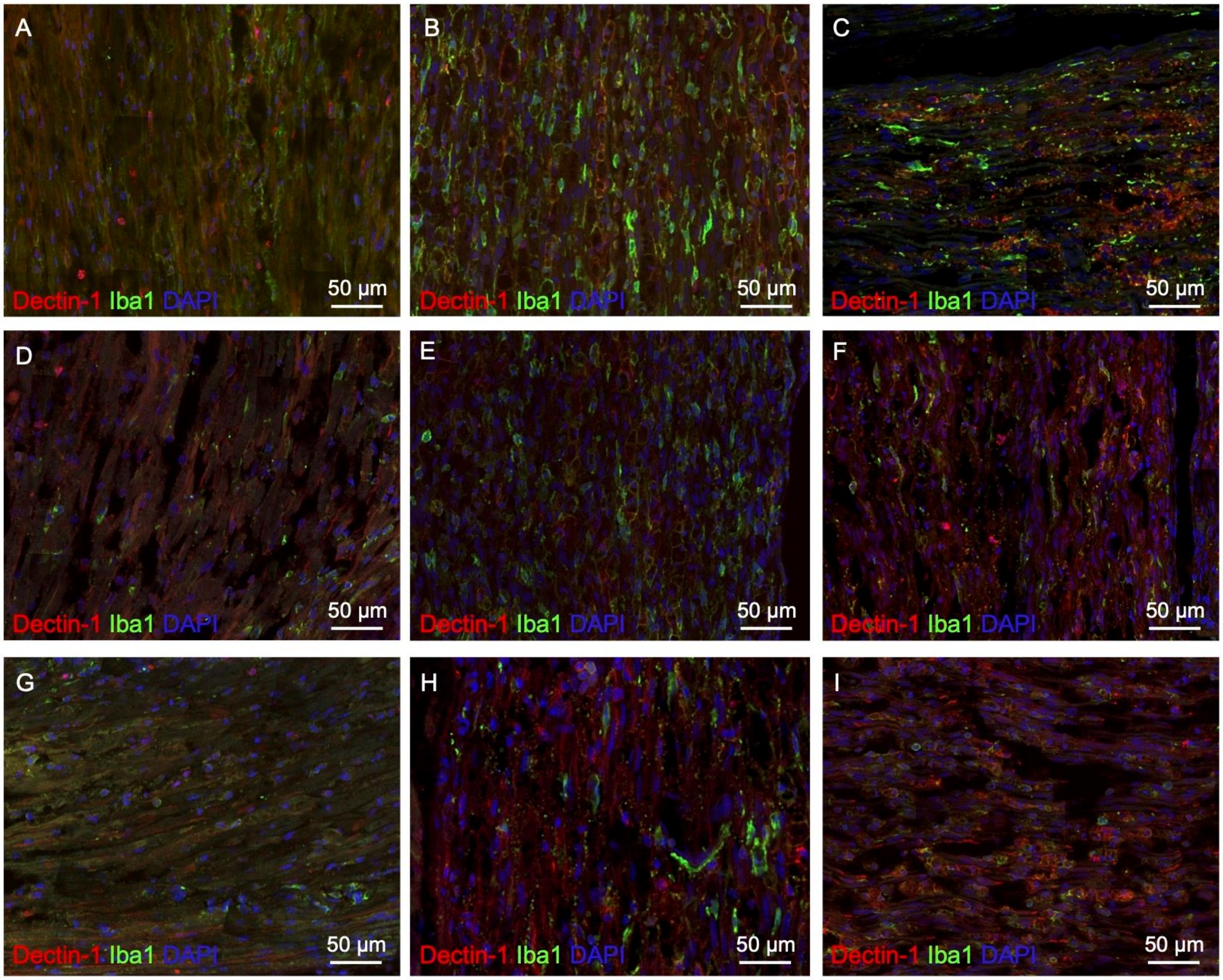

Supplementary Figure 1. Dectin-1 and Iba1 colocalization on crushed sciatic nerves. Longitudinal sections of the ipsilateral sciatic nerve from 500 μm proximal to 500 μm distal to the crushed site were stained with anti-Dectin-1 and anti-Iba1 antibodies. Representative figures are from (A) crushing injury plus PBS injection three days after injury, (B) crushing injury plus PBS injection seven days after injury, (C) crushing injury plus PBS injection 14 days after injury, (D) crushing injury plus WGPS injection three days after injury, (E) crushing injury plus WGPS injection seven days after injury, (F) crushing injury plus WGPS injection 14 days after injury, (G) crushing injury plus curdlan injection three days after injury, (H) crushing injury plus curdlan injection seven days after injury, (I) crushing injury plus curdlan injection 14 days after injury

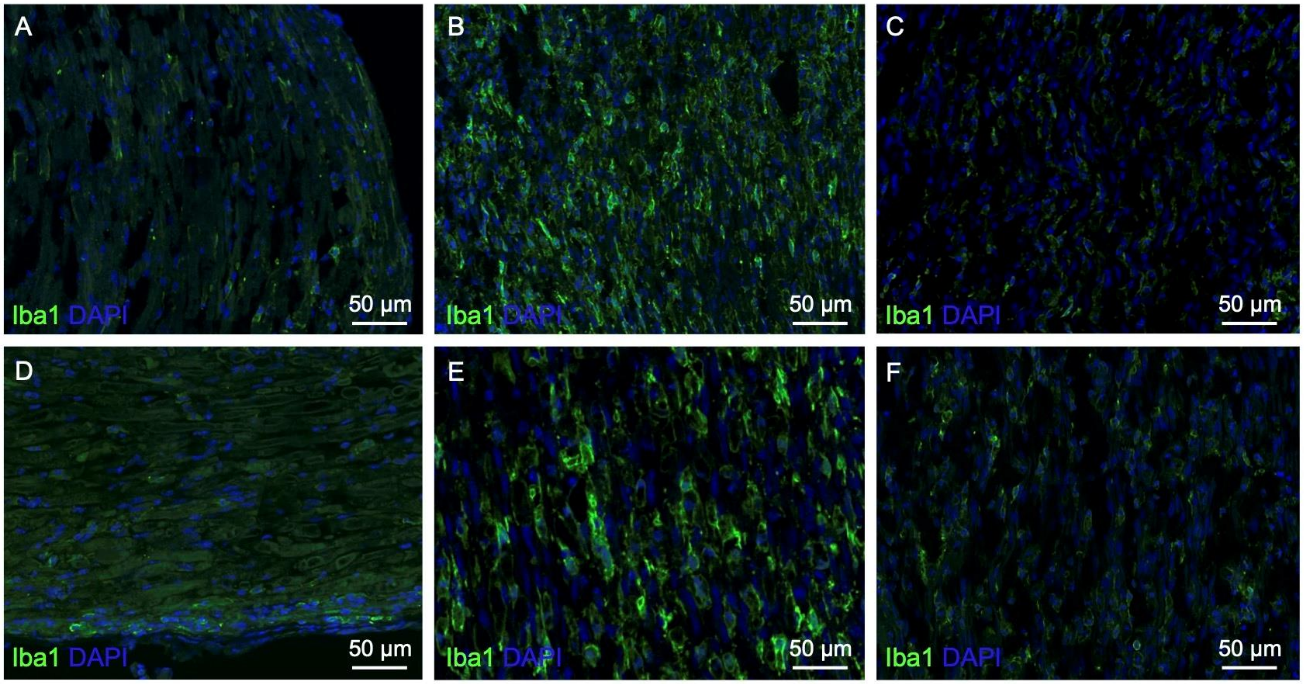

Supplementary Figure 2. Iba1 expression on crushed sciatic nerves. Longitudinal sections of the ipsilateral sciatic nerve from 500 µm proximal to 500 µm distal to the crushed site were stained with anti-Iba1 antibody. Representative figures are from (A) crushing injury plus WGPS injection three days after injury, (B) crushing injury plus WGPS injection seven days after injury, (C) crushing injury plus WGPS injection 14 days after injury, (D) crushing injury plus curdlan injection three days after injury, (E) crushing injury plus curdlan injection seven days after injury, (F) crushing injury plus curdlan injection 14 days after injury

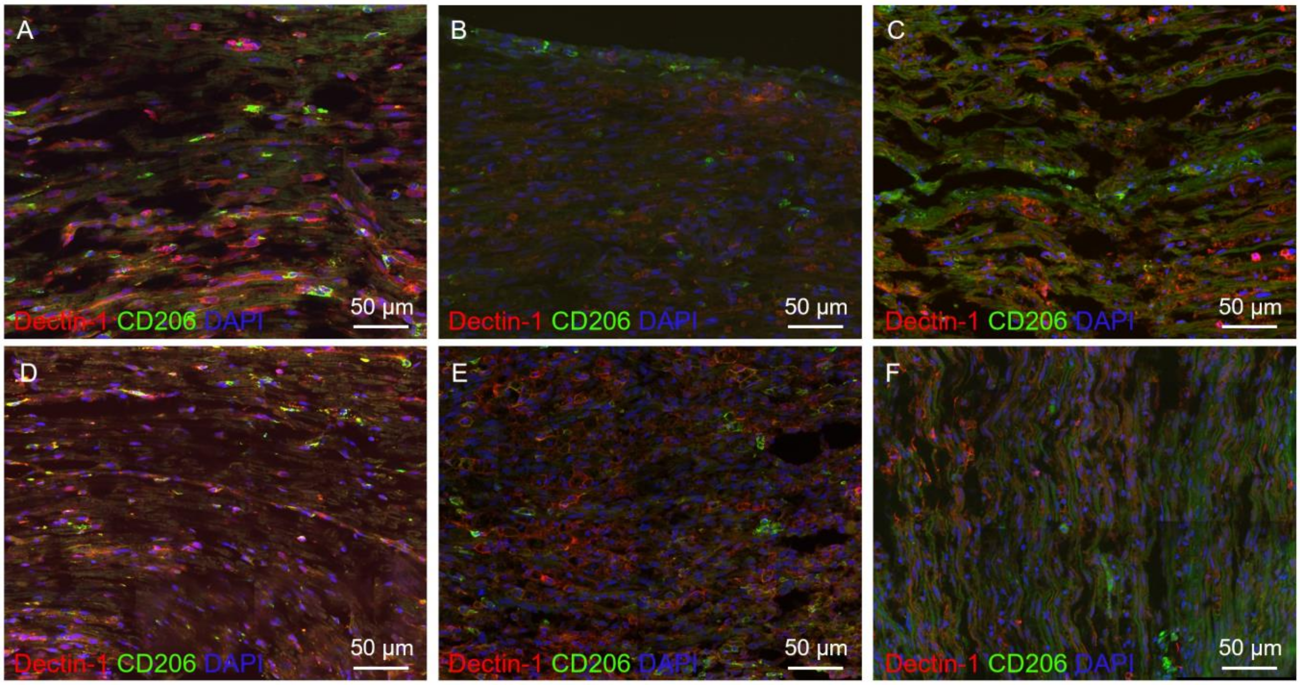

Supplementary Figure 3. Dectin-1 and CD206 colocalization on crushed sciatic nerves. Longitudinal sections of the ipsilateral sciatic nerve from 500 μm proximal to 500 μm distal to the crushed site were stained with anti-CD206 and anti-Dectin-1 antibodies. Representative figures are from (A) crushing injury plus WGPS injection three days after injury, (B) crushing injury plus WGPS injection seven days after injury, (C) crushing injury plus WGPS injection 14 days after injury, (D) crushing injury plus curdlan injection three days after injury, (E) crushing injury plus curdlan injection seven days after injury, (F) crushing injury plus curdlan injection 14 days after injury
